# Marker assisted improvement for leaf rust and moisture deficit stress tolerance in wheat variety HD3086

**DOI:** 10.3389/fpls.2022.1035016

**Published:** 2022-10-24

**Authors:** V.P. Sunilkumar, Hari Krishna, Narayana Bhat Devate, Karthik Kumar Manjunath, Divya Chauhan, Shweta Singh, Nivedita Sinha, Jang Bahadur Singh, T. L. Prakasha, Dharam Pal, M. Sivasamy, Neelu Jain, G. P. Singh, P. K. Singh

**Affiliations:** ^1^ Division of Genetics, Icar- Indian Agricultural Research Institute, New Delhi, India; ^2^ ICAR-Indian Institute of Wheat and Barley Research, Karnal, India

**Keywords:** *Lr24*, leaf rust resistance, drought tolerance, QTLs, MABB

## Abstract

There is a significant yield reduction in the wheat crop as a result of different biotic and abiotic stresses, and changing climate, among them moisture deficit stress and leaf rust are the major ones affecting wheat worldwide. HD3086 is a high-yielding wheat variety that has been released for commercial cultivation under timely sown irrigated conditions in the Indo-Gangetic plains of India. Variety HD3086 provides a good, stable yield, and it is the choice of millions of farmers in India. It becomes susceptible to the most prevalent pathotypes 77-5 and 77-9 of Puccinia triticina (causing leaf rust) in the production environment and its potential yield cannot be realized under moisture deficit stress. The present study demonstrates the use of a marker-assisted back cross breeding approach to the successful transfer of leaf rust resistance gene Lr24 and QTLs linked to moisture deficit stress tolerance in the background of HD3086. The genotype HI1500 was used as a donor parent that possesses leaf rust-resistant gene Lr24, which confers resistance against the major pathotypes found in the production environment. It possesses inbuilt tolerance under abiotic stresses with superior quality traits. Foreground selection for gene Lr24 and moisture deficit stress tolerance QTLs linked to Canopy temperature (CT), Normal Differential Vegetation Index (NDVI) and Thousand Kernel Weight (TKW) in different generations of the backcrossing and selection. In BC2F2, foreground selection was carried out to identify homozygous lines based on the linked markers and were advanced following pedigree based phenotypic selection. The selected lines were evaluated against P. triticina pathotypes 77-5 and 77-9 under controlled conditions. Recurrent parent recovery of the selected lines ranged from 78-94%. The identified lines were evaluated for their tolerance to moisture stress under field conditions and their resistance to rust under artificial epiphytotic conditions for two years. In BC2F5 generation, eight positive lines for marker alleles were selected which showed resistance to leaf rust and recorded an improvement in component traits of moisture deficit stress tolerance such as CT, NDVI, TKW and yield compared to the recurrent parent HD3086. The derived line is named HD3471 and is nominated for national trials for testing and further release for commercial cultivation.

## Introduction

Wheat (*Triticum aestivum* L.), the world’s most important food grain, is the staple food for over 27% of the global population in more than 40 countries (Sharma et al., 2019). It is generally known as the “King of Cereals” due to its high economic importance and is grown in a variety of agro-climatic conditions. The total wheat production in the world is 778.6 mt from an area of 220.4 mha with a productivity of 3.47 tons/ha (FAS; USDA, 2021). Indian wheat production is 107.86 mt obtained from an area of 31.45 mha with productivity of 3.37tons/ha (FAS; USDA, 2021). Various biotic and abiotic stresses affect the wheat crop, leading to significant yield reduction in annual wheat production. It is predicted that global warming will increase by 1.5°C in the next decade and annual precipitation will decrease by 4–27% in different parts of the world (IPCC, 2021). Drought and heat cause up to 86% and 69% yield losses in wheat, respectively (Prasad et al., 2011; Yang et al., 2021). Drought is the inadequacy of water availability including precipitation and soil moisture storage during the crop growth period both in duration and quantity which restrict the genetic yield potential (Sinha, 1986). It is recognized that half of the wheat cultivated in the developing world is sown under rain fed systems, which receive less than 600mm annual rainfall. In India, erratic distribution of rainfall and reduced groundwater table (Rodell et al., 2009) have adversely affected wheat production in major wheat growing zones.

Among the biotic factors that reduce wheat productivity, rusts are of prime importance. Rusts pathogens are continuously evolving and breaking the resistance of wheat cultivars. Prevailing diverse climatic conditions provide a conducive environment for epidemics of rust in one or other parts of the country. It is estimated that diseases reduce wheat yield by 15-20 percent. However, serious epidemics of leaf rust can reduce yield by up to 50%. Yield losses from leaf rust are mostly due to reductions in kernel weight. Leaf or brown rust caused by *Puccinia triticina* Eriks is probably the most important disease worldwide. There are currently 82 leaf rust resistance genes identified so far (McIntosh et al., 2017; Qureshi et al., 2018; Bariana et al., 2022) out of which *Lr1, Lr3, Lr9, Lr10, Lr13, Lr14a, Lr17, Lr19, Lr23, Lr24, Lr26, Lr28* and *Lr34* are commonly exploited in Indian wheat breeding programs (Bhardwaj et al., 2005; Bhardwaj et al., 2010, Bhardwaj et al., 2011). Among these leaf rust resistance genes, *Lr24*/*Sr24* derived from *Agropyron elongatum*, located on 3DL, confers resistance to all the currently prevalent pathotypes in the Indian sub-continent (Bhardwaj et al., 2021). Developing resistant wheat cultivars is the most efficient, economical, and environment friendly approach for the management of rusts.

Genetic variation for moisture deficit stress tolerance exists in wheat cultivars and improved adaptation response in wheat can be achieved by implementing appropriate crossing and selection strategies (Reynolds, 2001; Langridge and Reynolds, 2015). Earlier studies suggest that physiological traits associated with yield under drought have the potential to increase selection efficiency (Condon et al., 2004; Olivares-Villegas et al., 2007; Araus et al., 2008; Reynolds and Langridge, 2016). Molecular markers proved to be an important tool in improving selection efficiency and have good prospects for marker-assisted selection in improving moisture deficit stress in wheat (Kuchel et al., 2005; Quarrie et al., 2005). Physiological traits play crucial roles in determination of moisture stress tolerance hence incorporation of them using molecular marker in to the heigh yielding genotype can produce combination of high yielding and drought tolerant genotypes. SPAD meter is a portable diagnostic tool that measures leaf chlorophyll index *via* light transmittance that is differentially observed by chlorophyll and estimates leaf chlorophyll content and nitrogen content (Prasad et al., 2011), whereas a field-portable NDVI sensor enables quick ground-level measurements of crops with the resolution necessary to characterize the canopy for its biomass, nutrient content, and leaf area and green area indices (Weier and Herring, 2000; Araus et al., 2008). QTLs linked with canopy temperature govern the deeper root system to enhance the extraction of water from deeper soil horizons and also have been found to co-localize with regions affecting other drought adaption features like kernel number, grain yield, and chlorophyll content (Diab et al., 2008; Olivares-Villegas et al., 2008; Pinto et al., 2010; Pinto and Reynolds, 2015).

**Figure 1:**
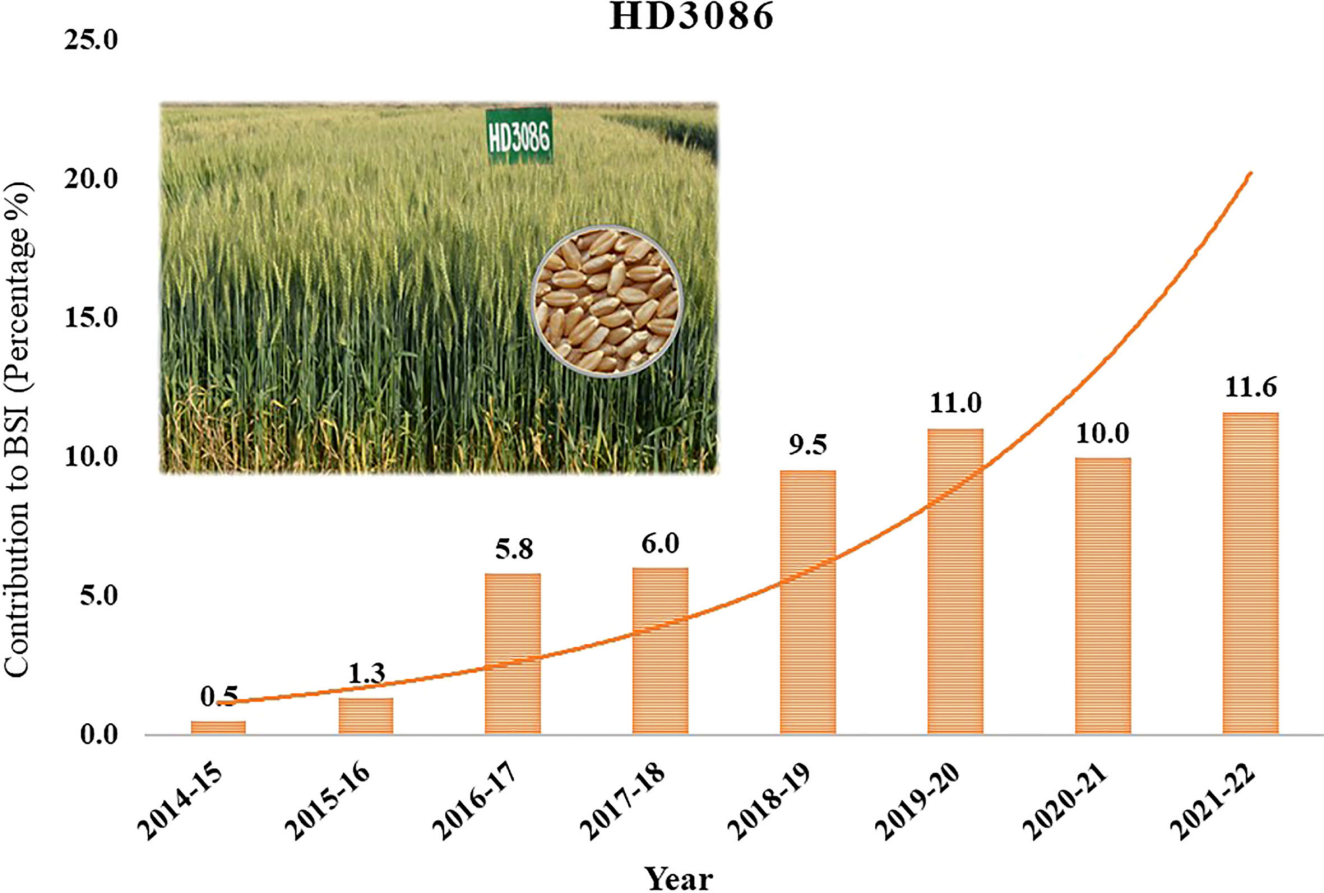
Yearly contribution of Wheat variety HD3086 to the total Breeder Seed Indent of country in percentage.

Indian Agricultural Research Institute (IARI) has a major contribution in developing high yielding wheat varieties in the interest of increasing the profit of farmers and wheat annual production in the country. IARI developed many high yielding wheat varieties, among them HD2967 and HD3086 together occupying 40% of the country’s total wheat area over the last several years. HD3086 (Pusa Gautami) is a high-yielding wheat variety that has been released for commercial cultivation under timely sown irrigated conditions in the North Western Plains Zone (NWPZ) and North Eastern Plains Zone (NEPZ) of the country. ‘HD3086’ alone occupies breeder seed indent of 34% among IARI wheat varieties and 11.6% of total wheat varieties indented in the country (https://seednet.gov.in/) (**Figure 1**). It is the choice of millions of farmers in the major wheat growing regions of the country, providing a good, stable yield over the years. Recent AICRIP reports (http://www.aicrpwheatbarleyicar.in) showed that HD3086 is susceptible to major pathotypes of *P. triticina* and recorded yield reduction under restricted irrigation conditions (AICRP Crop Improvement Reports, 2019). This is supported by Single Race Testing (SRT), in which HD3086 showed susceptibility to the prevalent 77 groups of pathotypes 77-5 and 77-9 of *P. triticina*. Hence, the improvement of HD3086 for leaf rust resistance and moisture deficit stress tolerance helps to expand the area of cultivation in the NWPZ, and NEPZ with a reduced number of irrigations. Because of the presence of the resistance gene *Lr24/Sr24*, the wheat variety HI1500 is promising in resistance against new pathotypes of *P. triticina*, 77-5 and 77-9. It is also known to perform well under limited irrigation conditions. Hence, HI1500 is used as a donor parent to transfer leaf rust resistance gene *Lr24/Sr24* and moisture deficit stress tolerance QTLs to improve HD3086.

## Materials and methods

### Plant materials and generation of improved lines

In this marker assisted backcross breeding (MABB) program, HD3086 was used as a recurrent parent and HI1500 as a donor parent. HD3086 has a semi-erect growth habit and matures in 140-145 days, providing an average yield of 5.46 t/ha under timely sown irrigated conditions. HI1500 is a popular variety grown in the Central Zone (CZ) under restricted irrigation conditions, giving an average yield of 2.2 t/ha. It has an erect growth habit, takes 130–134 days to mature, and shows resistance to leaf and stem rusts.

**Figure 2:**
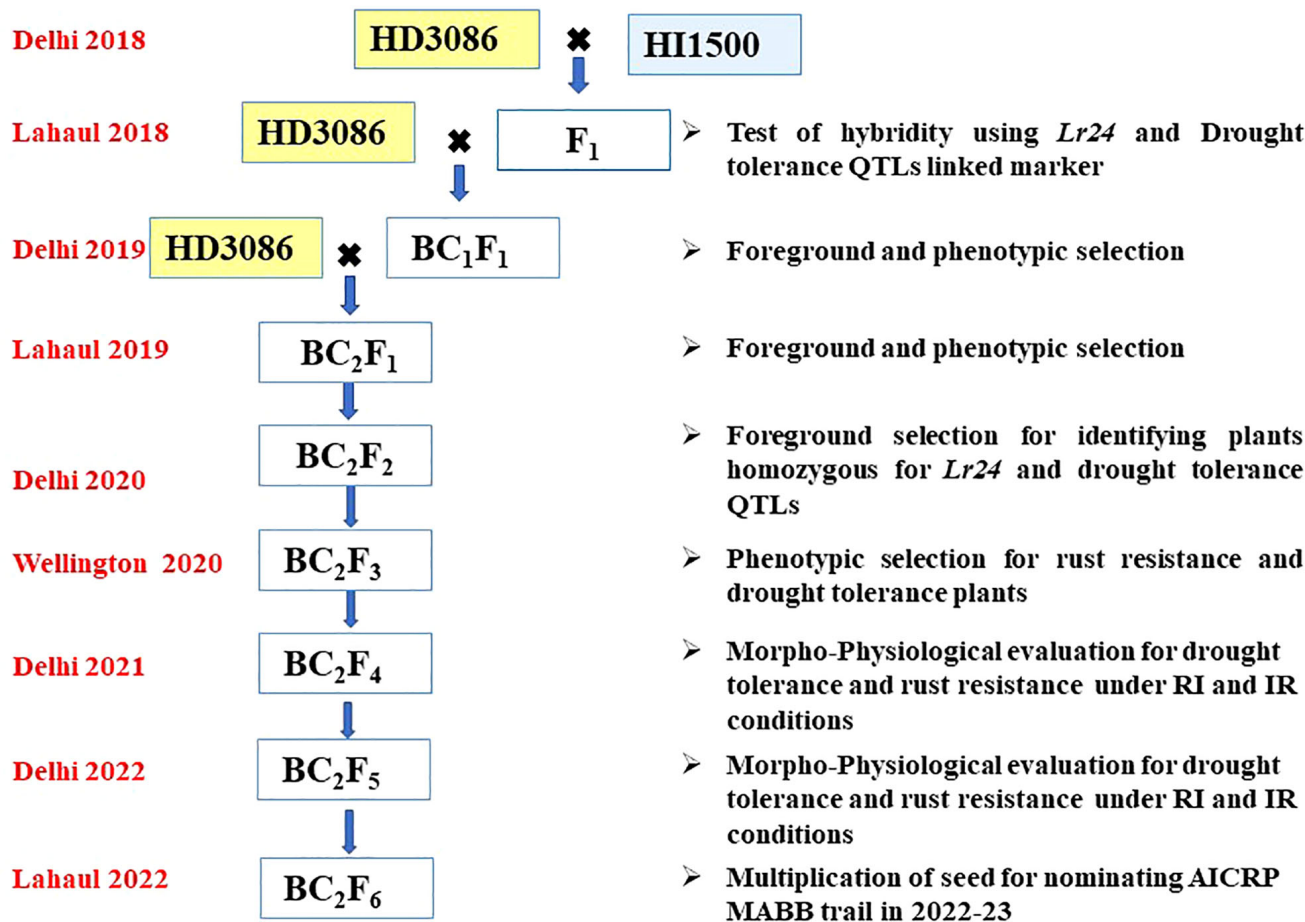
Schematic workflow of marker assisted backcross breeding of HD3086 × HI1500.

F_1_ plant was generated from the cross HD3086/HI1500, hybridity of F_1_ was confirmed using the *Lr24* linked SSR marker *Xbarc71*. F_1_ plant was backcrossed to the recurrent parent HD3086 to generate BC_1_F_1_. Foreground selection was carried out with SSR marker linked to leaf rust resistance gene (*Lr24*) and moisture deficit stress tolerance QTLs (**Table 1**), followed by phenotypic selection to identify the plants with maximum recovery for recurrent parent phenome (RPP). The best BC_1_F_1_ plants having heterozygote allele for rust resistance and moisture deficit stress tolerance QTLs along with visual similarity to the recurrent parent were selected and further backcrossed with HD3086 to generate BC_2_F_1_ seeds. In a similar way, the BC_2_F_1_ plants were also subjected to screening for *Lr24* and moisture deficit stress tolerance QTLs, and the superior positive plants were advanced to BC_2_F_2_ generation and foreground selection was repeated. Further, these BC_2_F_3_ (110 lines) were advanced *via* pedigree based phenotypic selection to BC_2_F_4_ (100 lines). The identified BC_2_F_4_ (100 lines) and BC_2_F_5_ (38) lines were evaluated for their tolerance to moisture stress under field conditions and their reactions to rust in artificial epiphytotic conditions for two years. The individuals in BC_2_F_5_ with superior performance were further advanced to multiplication of seed for nominating under MABB trails of AICRP (**Figure 2**).

**Table 1:** SSR and SCAR markers used in foreground selection linked to moisture deficit stress tolerant QTLs and leaf rust resistance genes *Lr24*.

SL.NO	Traits targeted	QTLs	PVE (%)	Gene/Marker used for Foreground selection	Reference
**1**	NDVI	*Qndvi4iari-2B*	14%, 19% (Harikrishna, 2017)	*Xcfd73*	Yang et al. (2007)
**2**	CT	*QCt_3A*(MQTL23) *Qct3.iari-5B*	11% (Harikrishna, 2017)	*Xbarc12- Xgwm369, Xgwm544*	Griffiths et al. (2009); Kumar et al. (2012); Gupta et al. (2012)
**3**	TKW	*Qtkw_2B*	–	*Xcfd73*	Yang et al. (2007), Liu et al. (2018)
**4**	Leaf rust resistance	*Lr24/Sr24*	–	SCAR: SCS1302, *Xbarc71*	Gupta et al. (2006); Pallavi et al. (2015)

### Foreground selection

Foreground selection was done according to the methods described by Hospital and Charcosset (1997) and Rai et al. (2018). QTLs linked to SSR markers associated with component traits of moisture deficit stress tolerance such as normalized difference vegetation index (NDVI), Leaf chlorophyll index (LCI), Canopy temperature (CT), and a yield contributing trait Thousand Kernal Weight (TKW) were selected from previously validated SSR markers linked to moisture deficit stress tolerance traits in HI1500 x DBW43 cross derived RIL population from our lab (Harikrishna, 2017). A positive allele for the parent HI1500, which is associated with component traits of moisture deficit stress tolerance viz., CT, NDVI, and TKW in the HI1500 x DBW43 RIL mapping population, was used for transfer Qct.iari_3A, linked to SSR *Xbarc12*, and Qndvi4.iari-2B, linked to SSR *cfd73*, explained a phenotypic variance of 14% (R2 = 0.14) at grain filling stage; Qct3.iari-5B linked to SSR *Xgwm544* explained a phenotypic variance of 11% (R2 = 0.11) at grain filling stage (Harikrishna, 2017) (**Table 1**). Foreground selection for leaf rust resistance was carried out with markers, *Lr24* (Schachermayr et al., 1995) and *Xbarc71* (Mago et al., 2005; Pallavi et al., 2015).

Genomic DNA from leaf samples was extracted using the CTAB method and DNA was quantified with Nanodrop, and quality was determined using 1% agarose gel electrophoresis with λ DNA as the standard. A total reaction volume of 15 µl was used for the PCR, which contained 40 ng of genomic DNA, 1X PCR buffer with 1.5 MgCl2, 10 pmol of each PCR primer, 100 µM of each dNTP, and 0.3 µl of Taq DNA polymerase. PCR Amplification of the template DNA was carried out in a ‘G-Storm thermal cycler’ with SSR and SCAR markers belonging to *Xgwm*, *Xbarc*, and *Xcfd* series (**Supplementary Table 1**). The amplified product was resolved in gel electrophoresis using 3% Agarose SFR gel and visualized on Gel documentation systems, GelDoc-It^®^² 315 (Supplier: UVP). Primer sequences for SSR markers were obtained from the Grain Genes website (http://wheat.pw.usda.gov/GG2/index.shtml) and synthesized for the Beltsville Agricultural Research Center (BARC), Gatersleben wheat microsatellite (GWM), Wheat Microsatellite Consortium (WMC), and INRA Clermont-Ferrand (CFD).

### Background selection

The selected homozygous BC2F5 lines screened for rust and moisture deficit stress tolerance linked markers, were genotyped using 35k SNPs from Axiom wheat breeders’ array along with the parents to identify plants with maximum recovery of recurrent parent genome (RPG). The recurrent parent HD3086 and donor HI1500 were analyzed for parental polymorphism and a total of 3707 polymorphic SNPs were selected. Graphical visualization of background recovery of the recurrent parent was done using software GGT 2.0 (Graphical Geno Typing 2.0) (Van Berloo, 2008). The contribution of the recurrent parent to the background of MABB derived lines was calculated by the formula:


G = [(B + 1/2A) × 100]/N

were,

N = total number of parental polymorphic markers screened

B = number of markers showing homozygosity for recurrent parent allele

A = number of markers showing heterozygosity for parental alleles.

### Screening for leaf rust resistance at seedling stage

The uredospore inoculum of individual pathotypes of *P. triticina*, 77-5 and 77-9 was obtained from the ICAR-IIWBR, Regional Station, Flowerdale, Shimla (India) for phenotyping of NILs. The seedlings were screened for resistance to *P. triticina* races 77-5 and 77-9 in each backcrossed generation during the main season at IARI New Delhi, IARI Regional Station, Indore and Shimla. In the off season, the material was evaluated for rust resistance at National Phytotron Facility (NPF), New Delhi. Seedlings, along with their parents, were inoculated with rust spores at the first leaf stage, about 8-10 days after sowing, during evening hours. The seedlings were sprayed with water to provide a uniform layer of moisture on the leaf surface before inoculation. Inoculated seedlings were incubated for 36 h in humid glass chambers at a temperature of 23 ± 2°C and relative humidity above 85%. Seedlings were shifted to muslin cloth chambers after incubation. The disease reaction was recorded 12-14 days after inoculation, using the scoring method described by Stakman et al. (1962).

### Screening of genotypes at adult stage for leaf rust resistance

Genotypes were screened at IARI New Delhi and IARI regional station, Indore, during the main season and at IARI regional station, Wellington, Tamil Nadu, during the off season. The rust susceptible landrace ‘Agra local’ was used as infector and was space planted after every twenty rows of test material and around the experimental plots. The rust disease scoring was done at the adult plant stage using the modified Cobb Scale (Peterson et al., 1948), which scores, S (susceptible), MS (moderately susceptible), MR (moderately resistant) and TR (trace) (Joshi et al., 1988) in the scale of 0 to 100.

### Evaluation for moisture deficit stress tolerance traits

The back cross populations were evaluated in the experimental farm of IARI, New Delhi (280 40’N,770 13’ E; MSL228m) during the year 2020-21and 2021-22. The positive BC2F4 lines having moisture deficit stress tolerance related QTLs along with the parents and 2 check varieties (GW322 and BABAX) were evaluated for agronomic and physiological traits in augmented design under restricted irrigation and irrigated condition. Lines were grown in 2 blocks containing 50 lines and four checks (2 parents and 2 checks) were replicated twice in each block. GW322 is the most common type of bread wheat in the central and peninsular zones, performs well in timely sown irrigated condition. Whereas BABAX, a drought tolerant Mexican cultivar perform well under water limited condition. Each BC2F4 line was planted in a plot containing three rows of 1m length with 23cm spacing between them, with a gross plot size of 0.63 m^2^. Superior lines were advanced to BC2F5 and were planted in a large plot size of 7.2 m^2^ (6m x 1.2m) Containing 6 rows. Standard agronomic management practices were followed for raising the wheat crop. The data was recorded on five plants from each of the entries for the agronomic characters such as thousand kernel weight (TKW), days to heading (DH), plant height (PH), grain yield (GY), grain weight per spike (GWPS) and grain length (GL). Physiological parameters such as NDVI (normalized difference vegetation index), CT (canopy temperature) and LCI (Leaf Chlorophyll Index), were recorded at 3 different stages such as vegetative stage (late boot stage, Z49), grain filling stage (early milk stage, Z73) and grain maturity stage (late milk stage, Z85) according to Zadoks scale (Zadoks et al., 1974).

### Statistical analysis

Calculation of descriptive statistics and analysis of variance was carried out in BC2F4 augmented RCBD, using the R program. BC2F5 derived lines sown in RBD with three replications were analyzed using data analysis add ins of MS-excel. Analysis of variance Critical Difference (CD) and Coefficient of Variation (CV) was calculated. Lines with statistical significance for moisture deficit stress tolerance with high yield under stress were identified.

## Results

### Development of backcross population following foreground selection and background analysis

The F1 plants obtained from the cross involving HI1500 (donor parent) and HD3086 (recurrent parent) were backcrossed with HD3086 to produce BC1F1 seeds. Among 120 BC1F1 plants, 16 were selected through foreground selection of QTLs associated with traits like CT, chlorophyll content, NDVI and TKW, and also for the *Lr24* gene associated with leaf rust resistance.

**Figure 3:**
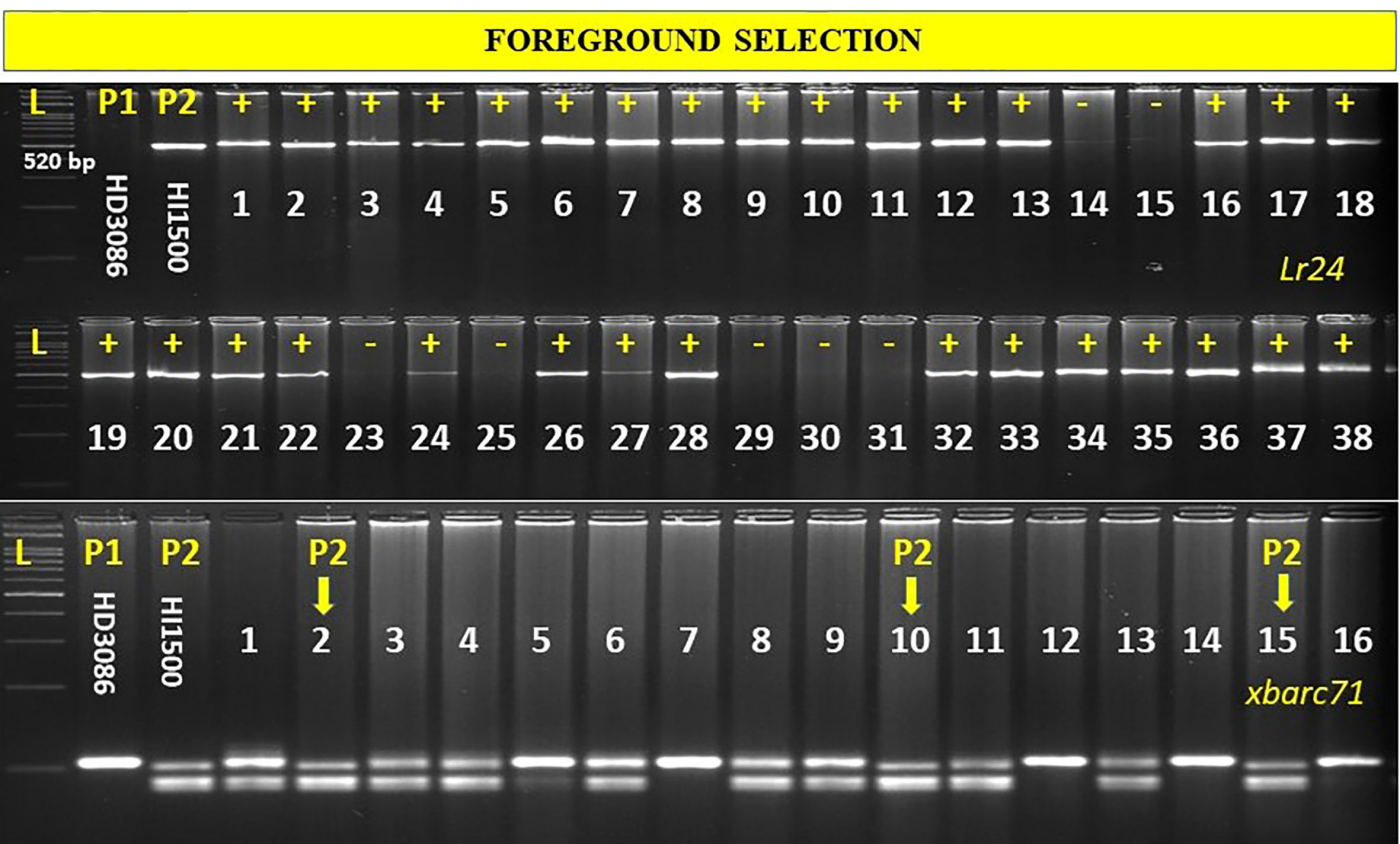
SCAR marker-assisted screening of the leaf rust resistance gene *Lr24* in selected BC_2_F_2_ lines and identity homozygous lines associated with *Lr24/Sr24* tightly linked SSR marker *Xbarc71*. In *Lr24* bands, progenies in the first row are 1-18 followed by second row from 19 to 38.

The selected plants for targeted traits were again backcrossed with the recurrent parent to generate BC2F1 plants. Out of 140 BC2F1 plants, 12 plants positive for a combination of moisture deficit stress tolerance QTLs and *Lr24* gene were selected and selfed to produce BC2F2 progenies (**Figure 3**). From these, a total of 600 BC2F2 plants were raised and subjected to stringent phenotyping of leaf rust screening for identification of homozygous plants **(** **Table 2**). Plants with rust resistance and good phenotyping background recovery were used for foreground selection using linked molecular markers to identify homozygous donor QTL combinations. From these, 110 BC2F2 plants were selected as homozygous for *Lr24*, and a combination of QTLs linked to moisture deficit stress tolerance using foreground selection. Further, these positive plants were sequentially advanced *via* pedigree based phenotypic selection until BC2F4.

**Table 2:** Number of plants selected in each generation in HD3086*2/HI1500 derived population.

Generation	No. of plants	Foreground Selection	
		No. of plants selected	
BC_1_F_1_	120	16	FS+PS
BC_2_F_1_	140	12	FS+PS
BC_2_F_2_	600	110	FS+PS
BC_2_F_3_	110	100	PS
BC_2_F_4_	100	38	BS+PS
BC_2_F_5_	38	14	PS
BC_2_F_6_	10	1	PS

FS, Foreground Selection; PS, Phenotypic Selection; BS, Background Selection.

### Screening for leaf rust resistance at seedling stage

The recurrent parent HD3086 showed a susceptible reaction (3 3+) for pathotypes 77-5 and 77-9 as that of susceptible check Agra local. On the other hand, donor parent HI1500 displayed an immune reaction (0) for pathotype 77-5 and resistance (; 1) for 77-9 (**Table 3**). In BC2F4, out of the 24 advanced lines subjected to SRT of *P. triticina* pathotypes 77-5 and 77-9, 19 lines showed resistance and 5 lines showed susceptible reaction due to the absence of *the Lr24* gene. The genotypes HD3086-E-5-17, HD3086-C-6-2 and HD3086-13-16 were identified as resistant/immune lines for both races 77-5 and 77-9 at IARI Regional Station, Indore. Whereas genotypes evaluated for SRT at Indore such as HD3086-M-1-26, HD3086-M-1-36, HD3086-I-3-1, HD3086-F-13-1 and HD3086-13-13 were classified as resistant for 77-5 and moderately susceptible for 77-9. The genotype HD3086-M-1-49 was identified as susceptible for 77-9 and moderately resistant for 77-5 (**Table 3**). Finally, nineteen (7HR, 10R and 2MR) BC2F4 lines showing resistance to pathotypes 77-5 and 77-9 and also performing better under restricted irrigation conditions were selected.

**Table 3:** Screening of marker assisted derived wheat genotypes for leaf rust races at IARI regional station Indore and Shimla (BC_2_F_4_).

Sl.no	Progenies	Rust race (77-5)		Rust race (77-9)		Reaction of genotypes
		IARI RS, Indore	IARI RS, Shimla	IARI RS, Indore	IARI RS, Shimla	
1	HD3086	33+	3+	3	3	Susceptible (S)
2	HI1500	0	0	; 1	0	Immune/Resistant (R)
3	HD3086-M-1-26	0	1	2	1	R
4	HD3086-M-1-36	; 1-	1	2	1	R
5	HD3086-M-1-49	0	2	2	0	MR
6	HD3086-M-1-55	2	1	1	;	R
7	HD3086-E-5-3	;	1	;	1	HR
8	HD3086-E-5-5	;	0	;	0	HR
9	HD3086-E-5-17	0	1	;	3	R
10	HD3086-E-5-26	3+	3	3	3	S
11	HD3086-E-5-27	3+	2	2	1	MS
12	HD3086-E-5-33	0	1	;	1	R
13	HD3086-E-5-43	;	0	;	1	HR
14	HD3086-E-5-60	0	;	;	0	HR
15	HD3086-C-6-1	3+	3	2 3	3	S
16	HD3086-C-6-2	0	1	; 1	0	R
17	HD3086-D-7-9	0	;	1	;	HR
18	HD3086-D-7-14	;	0	1	2	R
19	HD3086-D-7-56	;	1	;	1	R
20	HD3086-I-3-1	3	3	2 3	2 3	S
21	HD3086-F-13-1	0	;	2	2	R
22	HD3086-11-9	0	;	;	1	HR
23	HD3086-11-10	3+	2	3	3	S
24	HD3086-13-2	0	1	;	0	HR
25	HD3086-13-13	0	1	2	2	MR
26	HD3086-13-16	0	;	;	1	R
27	Agra Local	3+	3+	3+	3+	HS

HR, Highly resistant; R, Resistant; MR, Moderately resistant; MS, Moderately Susceptible; S, Susceptible; HS, Highly susceptible.

### Field screening for leaf rust resistance

Rust scoring in field conditions was carried out in BC2F1 (off season nursery: Lahul-Spiti), BC2F2 (Main Season: New Delhi) and BC2F3 (off season nursery: Wellington). In all these trials the MAS-derived *Lr24* selected lines showed a very low incidence of rust infection. Whereas recurrent parent HD3086 showed a severe disease infection (60S) of leaf rust. In BC2F5, out of 10 selected lines (previously selected in BC2F4 during SRT), 8 lines *viz*., HD3086-M-1-26-410-46, HD3086-M-1-36-413-47, HD3086-M-1-55-419-49, HD3086-E-5-17-461-50, HD3086-C-6-2-481-51, HD3086-F-13-1-512-53, HD3086-13-13-562-54 and HD3086-13-16-565-55 showed resistant reaction and lines HD3086-M-1-49-417-48 and HD3086-I-3-1-507-52 showed moderately resistant reaction. One MABB derived line, HD3086-C-6-2-481-51 showed ACI ‘0’ for leaf rust in initial plant pathological nursery data of AICRP trial 2021-22 (data available at http://www.aicrpwheatbarleyicar.in/wp-content/uploads/2022/07/PPSN-2021-22-decoded.pdf).

### Background analysis

**Figure 4:**
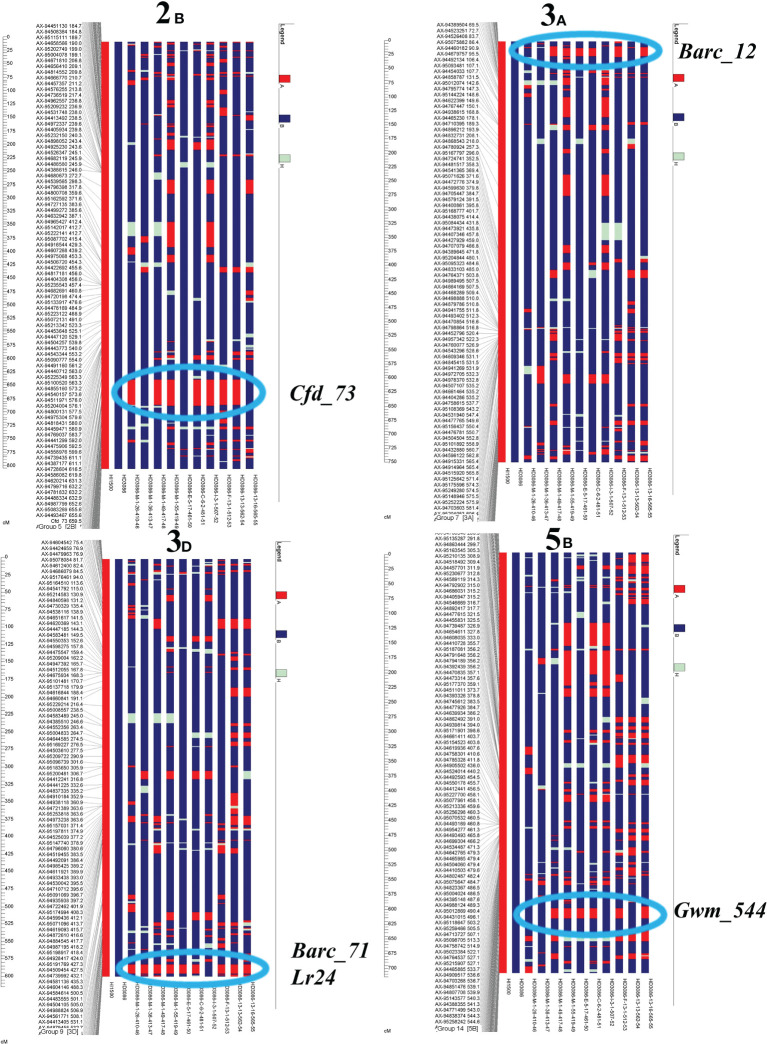
Recurrent parent recovery of chromosomes 2B, 3A, 3D and 5B.

Selected 10 best BC2F5 plants showed significant improvement over the recurrent parent HD3086, for component traits of moisture deficit stress tolerance and leaf rust resistance with maximum recovery of RPG with a range of 79%-95% and average RPG recovery, was 86%. Two superior lines HD3086-E-5-17-461-50 and HD3086-C-6-2-481-51 had maximum recovery of about 95.24% and 93.87% respectively (**Figure 4**; **Supplementary Table 4**).

### Morpho-physiological performance of the selected lines for moisture deficit stress tolerance

The phenotypic evaluation was performed in BC2F4 lines in augmented design (with 1m2 plot size) and 38 superior lines with rust resistance were advanced to BC2F5 for evaluation of yield and moisture deficit stress component traits in large plots (7.2m2) with RBD under irrigated (IR) and restricted irrigation (RI) conditions. The BC2F4 lines, HD3086-E-5-17-461-50 and HD3086-C-6-2-481-51 recorded the highest yield per plot followed by HD3086-M-1-26-410-46 and HD3086-F-13-1-512-53 during 2020-2021 under RI condition. The same lines advanced to BC2F5 showed consistent superior performance during the year 2021-22. The correlation study depicted that NDVI, LCI, TKW and GWPS were positively correlated with yield, while CT and DH were negatively correlated with yield under moisture deficit stress. MABB derived lines containing targeted QTLs were having significantly superior performance over the checks. All the 38 BC2F5 lines were evaluated under RI and IR conditions for distinctness, uniformity and stability (DUS) characterization. Eight lines are found to be consistent in performance during both the years and on par or superior to recurrent parent under moisture deficit stress with maximum phenotypic similarity to recurrent parent HD3086. All the superior 8 lines *viz*., HD3086-M-1-26-410-46, HD3086-M-1-36-413-47, HD3086-M-1-49-417-48, HD3086-M-1-55-419-49, HD3086-E-5-17-461-50, HD3086-C-6-2-481-51, HD3086-I-3-1-507-52, HD3086-F-13-1-512-53 were selected for subsequent entry and release (**Table 4**). The derived line HD3086-C-6-2-481-51 named HD3471, is nominated for national trials for testing and further release for commercial cultivation.

**Table 4:** Morpho-physiological characteristics of HD3086*2/HI1500 selected BC_2_F_5_ lines for component traits of moisture deficit stress tolerance under moisture deficit stress condition.

S.no	Selected progeny	QTLs linked trait	Leaf chlorophyll index (LCI)		Canopytemperature(°C)			NDVI			GL (mm)	DH(days)	GWPS (g)	TKW (g)	YLD (q/ha)	APR	Total% RPG %
			VS	GFS	VS	GFS	GMS	VS	GFS	GFS							
1	HD3086-M-1-26-410-46	NDVI+*Lr24*	44.5	51.8	28.3	31	35.3	0.78	0.66	0.42	6.84	88	1.22	34	45.01	R	88.31
2	HD3086-M-1-36-413-47	DH*+Lr24*	51.2	48.4	25.8	31.6	35.3	0.77	0.65	0.41	6.79	83	1.71	41.3	40.16	R	90.46
3	HD3086-M-1-49-417-48	TKW+CT+*Lr24*	49.2	54.2	27.5	32.1	35.5	0.76	0.65	0.4	7.11	92	1.41	39.4	42.24	MR	87.37
4	HD3086-M-1-55-419-49	CT+TKW+*Lr24*	50.5	51.6	26.4	32.5	35.4	0.78	0.64	0.42	7.27	91	1.39	43.2	36.7	R	82.74
5	HD3086-E-5-17-461-50	CT+Chl+*Lr24*	50.2	52.7	21.1	30.3	33.9	0.78	0.68	0.46	7.11	96	1.83	42.5	47.53	R	95.24
6	HD3086-C-6-2-481-51	CT+NDVI+TKW+*Lr24*	48.4	54.6	28	32.7	34.4	0.72	0.61	0.32	6.61	90	1.74	41	48.11	R	93.87
7	HD3086-I-3-1-507-52	NDVI+TKW	52.2	55.2	27	31.5	35.8	0.77	0.62	0.35	6.95	88	1.29	40.1	36.7	MR	81.71
8	HD3086-F-13-1-512-53	CT+ TKW+*Lr24*	45.5	47.9	26.9	30.6	36	0.75	0.6	0.37	6.65	88	1.95	33.2	44.87	R	83.97
9	HD3086-13-13-562-54	NDVI+*Lr24*	51.2	51.4	26.6	32	35.3	0.79	0.64	0.5	6.44	93	1.13	34.6	38.36	R	79.13
10	HD3086-13-16-565-55	CT+*Lr24*	52	54.2	28.8	32.4	34.8	0.79	0.63	0.49	7.09	100	1.22	33.9	42.93	R	82.33
	HD3086(RI)		46.3	51.4	29.1	31.3	36.8	0.78	0.61	0.32	7.01	92	0.9	33.3	34.34	S	
	HD3086(IR)		49.4	52.6	22	31.3	32	0.79	0.72	0.54	7.1	93	1.65	45.6	63.57	S	
	HI1500(RI)		50.2	50.4	27.9	30.5	35.7	0.77	0.65	0.4	7.18	86	1.5	36.1	37.82	R	
	HI1500(IR)		49.2	51.6	24.5	29	31.5	0.75	0.63	0.4	7.19	89	1.69	43.5	39.84	R	
	Mean		49.1	51.9	26.95	31.54	35.35	0.77	0.64	0.41	6.92	90.5	1.44	37.63	41.23		
	CD at 5%		2.42	2.49	0.97	1.65	1.56	0.04	0.02	0.02	0.31	4.35	0.06	1.64	2.25		

CD, Critical Difference; LCI, Leaf Chlorophyll Index; NDVI, Normalized Difference Vegetation Index; GL, Grain Length; GWPS, Grain Weight Per Spike; TKW, Thousand Kernel Weight LR, Leaf rust; YLD, Yield; APR, Adult Plant Resistance; R, Resistant; MR, Moderately resistant; S, Susceptible; RPG, Recurrent Parent Genome; RI, Rainfed; IR, Irrigated; VS, Vegetative stage; GFS, Grain Filling Stage; GMS, Grain Maturity Stage.

## Discussion

The wheat varieties which can grow well and produce better yields in moisture deficit/dry land areas along with resistance to biotic stresses such as leaf rust are the immediate priority of the wheat breeding program. Several severe rust epidemics have been recorded in India since the early 1800s due to cereal rust diseases (Joshi et al., 1977). An effective and desirable method would be combining conventional wheat breeding with MABB by introducing targeted genes or QTLs into superior wheat cultivars for overcoming such biotic and abiotic stresses (Collard and Mackill, 2008). MAS has been used frequently in bread wheat for introgression of traits such as disease resistance and quality improvement [(Barloy et al., 2007; Gupta et al., 2010; Kumar et al., 2010; Malik et al., 2015; Shah et al., 2017; Sharma et al., 2021)]. However, it has been used rarely to improve moisture deficit stress tolerance in wheat (Merchuk-Ovnat et al., 2016; Rai et al., 2018). In the current study, we developed improved lines with resistance to leaf rust and relatively higher grain yield under RI conditions by transferring leaf rust resistance gene *Lr24* and major QTLs linked to component traits of moisture deficit stress tolerance.

The component traits of moisture deficit stress tolerance such as NDVI, LCI, and TKW had positive correlation and traits like CT and DH showed negative correlation with grain yield under the current investigation as previously reported (Lopes and Reynolds, 2012; Harikrishna et al., 2016; Ramya et al., 2016). Therefore, component traits of moisture deficit stress tolerance such as NDVI, leaf chlorophyll index, and canopy temperature strategically coupled with yield had a complementing influence on productivity under moisture deficit stress.

### Marker-assisted selection for leaf rust resistance gene *Lr24*

Leaf rust resistance gene *Lr24*, derived from *Thinopyrum elongatum*, confers resistance right from the seedling stage and is associated with the stem rust resistance gene *Sr24* (McIntosh et al., 1976; Singh et al., 2006). Therefore, the *Lr24/Sr24* confers resistance to the most prevalent leaf and stem rust races in India (Singh et al., 2017). Although virulence on *Lr24* has been reported in South Africa, North America, and South America, it still provides effective resistance in India. The first wheat variety containing *Lr24* was released in 1993 and since then, a number of varieties were released with *the Lr24* gene (Tomar et al., 2014). Hence, the presence of *Lr24* in Indian wheat varieties is extremely useful for resistance against leaf rust (Pal et al., 2022). In recent years, new virulent races have appeared against some leaf rust resistance genes (Prasad et al., 2019) among which 77-9 and 77-5 are most prevalent and virulent. The pathotype, 77-9 was found in 149 rust samples (51.1%), followed by pathotype 77-5 in 15.1% of the wheat leaf rust (Pt) samples collected from India and Nepal. The pathotype 77-9, which is virulent to *Lr37*, was unable to overcome the resistance of many varieties due to the presence of *Lr24* (Tinku Gautam et al., 2020b).

The traditional backcross strategy for transferring rust resistance genes needs discriminating races to be screened at the seedling or adult stage under epiphytotic circumstances. With the advent of genetic markers linked to resistance genes, it is possible to precisely identify the targeted rust resistance gene(s) in segregating populations (Sivasamy et al., 2009; Revathi et al., 2010; Bhawar et al., 2011). The polymorphic SCAR markers were developed for *Agropyron elongatum* derived leaf rust resistance gene *Lr24* and utilized in marker assisted selection (Gupta et al., 2006). Due to the dominant nature of the SCAR marker, identification of homozygous plants with *Lr24* in BC2F2 is not possible. Hence, it needs progeny testing in the BC2F3 generation to identify homozygous plants. In addition to this, specific microsatellite markers, *Xgwm114* and *Xbarc71*, were developed for *the Lr24* locus to select homozygous plants for leaf rust resistance at early generations (Pallavi et al., 2015) such as BC1F1, BC2F1, and BC2F2 generations. Therefore, we used codominant SSR marker *Xbarc71* in addition to *Lr24/Sr24* SCAR marker to select homozygous plants for *Lr24* in BC2F2. Homozygous lines for *Lr24* were selected in BC2F2 generation along with the phenotypic selection at each backcross generation for leaf rust in the seedling stage (SRT) and in the adult plant stage. Along with MAS, the phenotypic section for targeted traits increases the efficiency of selection (Davies et al., 2006). We developed 19 resistant (7 HR+ 10 R+2 MR) lines in BC2F4 generation against 77-5 and 77-9 races of leaf rust at two different locations. Similarly, leaf rust resistance genes *Lr19* and *Lr24* derived from *Thinopyrum* (syn. *Agropyron*) were transferred into superior wheat cultivar HD2733 through marker-assisted selection (Singh et al., 2017). The genomic segment carrying the *Lr24*/*Sr24* genes also resulted in improved grain quality without any yield penalty in HD2733 suggesting the added gains of utilizing this region for improving grain quality besides leaf rust resistance (Rai et al., 2021). In another study, major QTL for post-harvest sprouting tolerance and *Lr* genes such as *Lr24 + Lr28* were transferred into a cultivar HD2329 using MAS against virulent pathotype 77-5 (Kumar et al., 2010).

### Marker-assisted selection for component traits of moisture deficit stress tolerance

tBreeding for complex traits like moisture deficit stress tolerance is a challenging task with conventional breeding methods due to the inheritance of polygenes and with relatively low heritability and high environmental influence (Talukdar et al., 2014; Ramya et al., 2016). Therefore, molecular markers assisted breeding is a better option to develop moisture deficit stress tolerance along with disease resistance. Numerous studies have revealed QTLs for grain yield and component traits of moisture deficit stress tolerance (Pinto et al., 2010; Gupta et al., 2012; Gupta et al., 2017; Shashikumara et al., 2020), which can be used in practical breeding with MABB for an effective and desirable way to introduce them into superior wheat cultivars (Collard and Mackill, 2008). CT, NDVI, LCI, and TKW are thought to be complementary traits to yield because they enhance wheat yield under moisture deficit through stress tolerance mechanisms (Lopes and Reynolds, 2012). Therefore, in this study 3 QTLs linked to component traits of moisture deficit stress tolerance (NDVI and TKW: *Xcfd73*, CT and Leaf chlorophyll index: *Xbarc12*, CT: *Xgwm544)* have been successfully transferred in the genetic background of HD3086 through MABB along with phenotypic selection. These three QTLs under transfer were validated in RIL population HI1500 x DBW43 by Harikrishna (2017). In past, a few studies have introduced QTLs into wheat to enhance drought tolerance and yield under moisture deficit conditions (Merchuk-Ovnat et al., 2016; Rai et al., 2018; Gautam et al., 2020a; Todkar et al., 2020).

**Figure 5:**
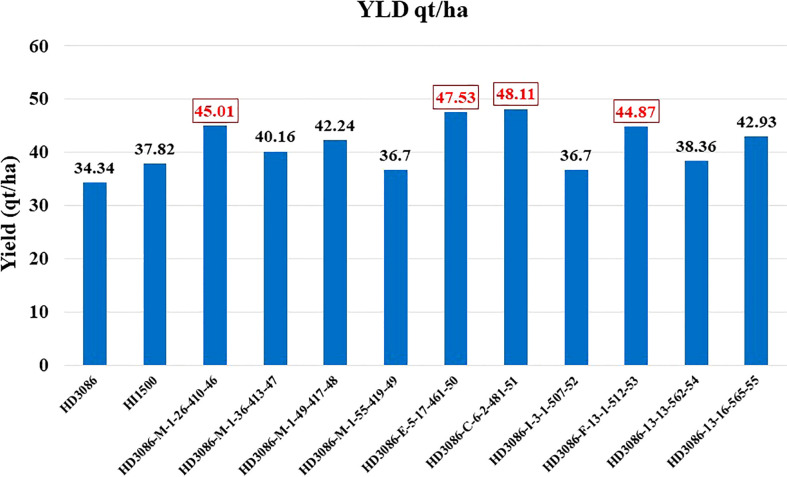
Improvement in grain yield of selected lines of HD3086*2/HI1500 derived BC_2_F_5_ population under moisture deficit stress.

Foreground selection was undertaken in BC1F1, BC2F1 and BC2F2 for the successful transfer of three QTLs in combination linked to component traits of moisture deficit stress tolerance in the present study. The four lines having better grain yield of 30-40% and best yield improvement observed in line having QTLs combination linked to traits NDVI, CT and TKW along with leaf rust resistance over recurrent parent HD3086 were developed and proposed to the varietal trial evaluation program (**Figure 5**). In a similar previous study by Rai et al. (2018) five potential varieties performing well under rain fed conditions were developed in the background of HD2733 lines by transferring NDVI, CT and chlorophyll content linked QTLs through MABB. Similarly, variety GW322 was improved for traits NDVI, stay green, chlorophyll content and yield through MABB and 18 superior BC2F3 moisture deficit stress tolerant progenies were identified (Todkar et al., 2020).

It is challenging to cover entire genomic regions with evenly dispersed polymorphic markers due to the large genome size of wheat (~16GB; Somers et al., 2004). To speed up the RPG recovery in MABB, background selection utilizing polymorphic markers with genome-wide coverage has been widely adopted (Chen et al., 2008; Basavaraj et al., 2010). SNPs would be a preferable option for the background selection in MABB because the background analysis using SNP assay was over 300 times more cost-effective than SSR markers (Khanna et al., 2015). We performed background analysis of selected BC2F4 lines using 35k SNPs to identify the plant with maximum recurrent parent genome and obtained a recovery range of selected lines from 79 to 95% with an average recovery of 86%. Two selected lines with positive *Lr24* allele *viz*., HD3086-C-6-2-481-51and HD3086-E-5-17-461-50 were having a recovery of about 95.24% and 93.87% respectively. Similarly, 94.55% genome recovery in BC2 generations was observed during pyramiding of leaf rust resistance genes into an elite cultivar HD2687 (Bhawar et al., 2011) and genome recovery of 89.2%–95.4% for drought tolerance QTLs into HD2733 by Rai et al. (2018) in MABB studies.

## Conclusion

The current study is on the improvement of wheat cultivar for both biotic and abiotic stresses in combination using MABB and phenotypic selection in wheat. We have transferred *Lr24*, and 3 QTLs linked to component traits of moisture deficit stress tolerance using MABB to develop improved lines for resistance to leaf rust and moisture deficit stress. The improved lines had comparable grain yield production to the original parent, HD3086 along with high chlorophyll content, low canopy temperature, high normalized difference vegetation index, and other favorable morpho-physiological characteristics under moisture deficit stress. Wheat variety HD3086 is a choice of millions of farmers in major wheat growing regions because of its high adaptability and quality. Improvement of other traits of this variety such as disease resistance and moisture deficit stress tolerance help in expanding the area of cultivation with a limited number of irrigations.

## Data availability statement

The raw data supporting the conclusions of this article will be made available by the authors, without undue reservation.

## Author contributions

PS, GS, and NJ designed and supervised the conduct of experiments and provided critical inputs. SP performed experiments. SP, SS, DC, ND, and JS collected phenotypic data. SP, HK, PL, DP, and SM evaluated lines for rust resistance. SP, PS, HK, SS, DC, NS, and KM contributed in the generation of genotyping data. SP, ND, and HK did the statistical analysis. SP, HK, and NJ prepared the manuscript. All authors contributed to the article and approved the submitted version.

## Funding

The part of this research supported by the grant from Bill and Melinda Gates Foundation (OPP1194767) for SNP genotyping and funding from ICAR-NICRA project.

## Acknowledgments

SP acknowledges the ICAR-Indian agriculture research institute (IARI), New Delhi for provided scholarships to complete this work as part of Ph.D. thesis.

## Conflict of interest

The authors declare that the research was conducted in the absence of any commercial or financial relationships that could be construed as a potential conflict of interest.

## Publisher’s Note

All claims expressed in this article are solely those of the authors and do not necessarily represent those of their affiliated organizations, or those of the publisher, the editors and the reviewers. Any product that may be evaluated in this article, or claim that may be made by its manufacturer, is not guaranteed or endorsed by the publisher.
