# Supplementary material for: Marker assisted improvement for leaf rust and moisture deficit stress tolerance in wheat variety HD3086
Source: Front Plant Sci. 2022 Oct 24;13:1035016. doi: 10.3389/fpls.2022.1035016 (PMC9638138; doi:10.3389/fpls.2022.1035016)
Supplement: Supplementary file 1 [file DataSheet_1.docx]

**SUPPLEMENTARY TABLES**

**Supplementary Table 1: Sequence of STS and SSR primers and their Annealing temperature (°C) for detection of STS and SSR markers for resistance genes *Lr24* and Drought QTLs in HD3086 derived wheat genotypes**

| **SL.NO** | **Gene/Marker** | **Chromosome** | **Primer sequence (5’ – 3’)** | **Annealing temperature (°C)** | **Associated Traits** | **Reference** |
| --- | --- | --- | --- | --- | --- | --- |
| **1** | *Lr24*  SCAR: SCS1302 | 3DL | F: CGCAGGTTCCAATACTTTTC  R: CGCAGGTTCTACCTAATGCAA | 60 | Leaf rust | Gupta et al. (2006) |
| **2** | *Xbarc71* | 3D | F - GCGCTTGTTCCTCACCTGCTCATA  R -GCGTATATTCTCTCGTCTTCTTGTTGGTT | 55 | Leaf rust | Mago et al. (2005), Pallavi et al (2015) |
| **3** | *Xcfd73* | 2B | F - GATAGATCAATGTGGGCCGT  R - AACTGTTCTGCCATCTGAGC | 60 | NDVI | Yang et al. (2007) |
| **4** | *Xbarc12* | 3A | F - CGACAGAGTGATCACCCAAATATAA  R - CATCGGTCTAATTGTCAATGTA | 52 | CT(MQTL23) | Kumar et al. (2012) |
| **5** | *Xbarc186* | 5A | F - GGAGTGTCGAGATGATGTGGAAAC  R- CGCAGACGTCAGCAGCTCGAGAGG | 58 | CT, NDVI, DH, TKW | Pinto et al. (2010) |
| **6** | *Xgwm369* | 3A | F - CTGCAGGCCATGATGATG  R - ACCGTGGGTGTTGTGAGC | 60 | CT | Kumar et al. (2012) |
| **7** | *Xgwm544* | 5B | F - TAGAATTCTTTATGGGGTCTGC  R - AGGATTCCAATCCTTCAAAATT | 55 | Grain length, CT | Griffith *et al.* (2009) |

| Years | Stem rust | | Leaf rust (S) | | Leaf rust (N) | | Yellow rust(N) | |
| --- | --- | --- | --- | --- | --- | --- | --- | --- |
|  | ACI | HS | ACI | HS | ACI | HS | ACI | HS |
| 2018-19 | 26.7 | 40S | 10.8 | 60S | 28.1 | 40S | 10 | 40S |
| 2019-20 | 31 | 60S | 19.5 | 80S | 22.5 | 50S | 14.3 | 60S |
| 2020-21 | 30.8 | 80S | 21.3 | 60S | 19.3 | 60S | 9.5 | 40S |
| 2021-22 | 19.9 | 40S | 10.4 | 20S | 27.9 | 60S | 18.8 | 60S |

**Supplementary Table 2: Initial plant pathological nursery data of AICRP of HD3086**

**Supplementary Table 3: Initial plant pathological nursery data of AICRP on Wheat 2021-22 (Data source: http://www.aicrpwheatbarleyicar.in/wp-content/uploads/2022/07/PPSN-2021-22-decoded.pdf**

| Entry no | Stem rust | | Leaf rust (S) | | Leaf rust (N) | | Yellow rust(N) | |
| --- | --- | --- | --- | --- | --- | --- | --- | --- |
|  | ACI | HS | ACI | HS | ACI | HS | ACI | HS |
| HD3086 | 19.9 | 40S | 10.42 | 20S | 27.9 | 60S | 18.8 | 60S |
| Infector | 80 | 100S | 80 | 100S | 70 | 80S | 77.5 | 100S |
| HD3086-C-6-2-481-51 | 4 | 20S | 1.2 | 5S | 0 | 0 | 5.6 | 20MS |

**Supplementary Table 4: Chromosome wise recurrent parent genome recovery in HD3086*2/HI1500 selected BC_2_F_5_ lines**

|  | Recurrent parent genome recovery (%) | | | | | | | | | |
| --- | --- | --- | --- | --- | --- | --- | --- | --- | --- | --- |
| Chromosome | HD3086-M-1-26-410-46 | HD3086-M-1-36-413-47 | HD3086-M-1-49-417-48 | HD3086-M-1-55-419-49 | HD3086-E-5-17-461-50 | HD3086-C-6-2-481-51 | HD3086-I-3-1-507-52 | HD3086-F-13-1-512-53 | HD3086-13-13-562-54 | HD3086-13-16-565-55 |
| 1A | 94.58 | 96.08 | 84.64 | 84.64 | 96.69 | 95.78 | 82.53 | 87.65 | 87.95 | 87.35 |
| 1B | 92.46 | 87.07 | 91.81 | 80.60 | 98.28 | 98.06 | 80.60 | 91.16 | 87.07 | 84.27 |
| 1D | 96.33 | 95.18 | 92.89 | 78.21 | 96.56 | 94.04 | 76.83 | 83.26 | 83.03 | 81.19 |
| 2A | 92.53 | 85.75 | 92.31 | 66.74 | 94.80 | 96.61 | 66.29 | 89.14 | 80.77 | 73.08 |
| 2B | 90.19 | 92.45 | 89.06 | 76.98 | 96.79 | 91.51 | 75.28 | 81.89 | 87.74 | 83.58 |
| 2D | 91.05 | 90.17 | 91.27 | 79.04 | 96.72 | 95.41 | 77.73 | 89.52 | 80.13 | 82.10 |
| 3A | 89.79 | 91.62 | 78.27 | 75.39 | 91.10 | 88.74 | 79.32 | 67.80 | 80.10 | 76.44 |
| 3B | 75.85 | 96.59 | 85.61 | 93.17 | 95.85 | 96.59 | 90.98 | 81.95 | 69.51 | 82.68 |
| 3D | 80.23 | 86.72 | 81.36 | 80.51 | 91.53 | 88.98 | 85.03 | 74.01 | 72.32 | 78.25 |
| 4A | 78.75 | 81.25 | 80.63 | 85.00 | 93.13 | 94.38 | 80.00 | 86.25 | 75.63 | 75.63 |
| 4B | 93.06 | 91.67 | 93.75 | 85.42 | 92.36 | 95.83 | 83.33 | 90.97 | 76.39 | 83.33 |
| 4D | 96.03 | 87.30 | 91.27 | 90.48 | 95.24 | 94.44 | 88.10 | 90.48 | 87.30 | 88.89 |
| 5A | 91.62 | 92.20 | 90.46 | 79.48 | 94.51 | 89.02 | 78.32 | 86.99 | 75.43 | 84.39 |
| 5B | 93.65 | 92.06 | 87.70 | 70.04 | 93.85 | 90.87 | 73.61 | 77.98 | 65.67 | 72.22 |
| 5D | 92.15 | 91.28 | 85.76 | 84.01 | 95.06 | 93.90 | 82.85 | 82.56 | 75.29 | 80.23 |
| 6A | 96.00 | 86.80 | 84.80 | 87.60 | 96.00 | 94.80 | 85.20 | 82.80 | 81.60 | 81.60 |
| 6B | 95.48 | 92.46 | 93.47 | 84.67 | 97.24 | 95.98 | 83.42 | 87.44 | 88.94 | 90.20 |
| 6D | 96.88 | 91.02 | 90.23 | 82.42 | 95.70 | 92.58 | 80.08 | 89.84 | 73.05 | 83.98 |
| 7A | 77.39 | 90.20 | 75.13 | 91.21 | 96.48 | 96.73 | 88.44 | 79.90 | 70.60 | 85.43 |
| 7B | 64.67 | 91.85 | 90.22 | 91.58 | 96.20 | 94.57 | 88.59 | 74.46 | 81.52 | 90.49 |
| 7D | 75.96 | 90.06 | 84.29 | 90.38 | 96.15 | 92.63 | 89.42 | 87.50 | 81.73 | 83.65 |
| Total Recovery | 88.31614 | 90.46622 | 87.3766 | 82.74159 | 95.24886 | 93.87839 | 81.71206 | 83.97836 | 79.13234 | 82.33281 |
